# Relationship between muscle quality index and urinary incontinence among U.S. population: evidence from NHANES 2011 to 2014

**DOI:** 10.3389/fendo.2025.1533617

**Published:** 2025-04-08

**Authors:** Yiwang Hu, Hanyan Xu, Shuying Xie, Chengshui Chen, Xiong Lei

**Affiliations:** ^1^ Department of Colorectal Surgery, The First Affiliated Hospital of Wenzhou Medical University, Wenzhou, China; ^2^ Department of Pulmonary and Critical Care Medicine, The First Affiliated Hospital of Wenzhou Medical University, Wenzhou, China; ^3^ Department of Emergency, The First Affiliated Hospital of Wenzhou Medical University, Wenzhou, China

**Keywords:** muscle quality index, urinary incontinence, NHANES, cohort study, epidemiology

## Abstract

**Background:**

Urinary incontinence (UI) is a common and troublesome global problem. The purpose was to explore the relationship between muscle quality index (MQI) and UI.

**Methods:**

We performed a secondary analysis of the National Health and Nutrition Examination Survey (NHANES) database (2011 to 2014). Weighted logistic regression was used to analyze the relationship between MQI and UI. Subgroup analyses were further conducted to investigate the relationship. The *P* for trend and *P* for interaction were also conducted.

**Results:**

A total of 2,779 participants were enrolled in the study, comprising 1,241 females and 1,538 males with a median age of 36 years. The prevalence of UI was approximately 25.45%. In adjusted model, weighted multivariate logistic regression analyses showed that MQI was significantly negatively associated with UI (OR,0.65; 95%CI,0.50 to 0.85). Furthermore, the results revealed that the highest MQI group had a 33% reduction in UI compared to the lowest MQI group and the P for trend was less than 0.05. In subgroup analysis, the MQI was negatively associated with UI in females (OR, 0.64; 95%CI, 0.45 to 0.92), under 40 years old (OR,0.65; 95%CI,0.50 to 0.85), poverty-to-income ratio of 1 to 3 (OR, 0.48; 95%CI, 0.29 to 0.78), and Non-Hispanic Black (OR, 0.50; 95%CI, 0.29 to 0.87), and in some populations without hypertension or diabetes.

**Conclusion:**

The study revealed that a higher MQI was associated with a lower prevalence of urinary incontinence. This study provides insights into potential preventive strategies for UI.

## 1 Introduction

The prevalence of urinary incontinence (UI) is quite high in the U.S. population. The prevalence of UI is increasing annually in both men and women, with a particularly sharp rise observed in men as they age (1). The UI is associated with an increased risk of mortality related to cancer and cardiovascular diseases (2). In addition, UI was positively associated with depression (3). Therefore, the prevention of UI should be emphasized among the U.S. population.

Recently, many studies have focused on the relationship between muscle and handgrip strength with human disease. For example, sarcopenia was negatively correlated with the degree of recovery of voiding function during recovery from the disease (4). Low muscle mass increases the risk of death from cardiovascular disease in diabetics (5). Similarly, low handgrip strength is associated with an increased risk of cardiovascular mortality (6). Handgrip strength has been shown to have a significant correlation with urinary incontinence in the elderly (7). Pelvic floor muscle strength is positively correlated with handgrip strength and plays an important role in female UI (8). A higher percentage of muscle-to-fat ratio may provide a protective factor against female urinary incontinence (9). Therefore, assessing the association between muscle/grip strength and UI seems promising.

An indicator that combines handgrip strength (HGS) and muscle mass is called the MQI, which is defined as handgrip strength divided by the appendicular skeletal muscle mass (ASM) (10). MQI showed a negative correlation with depression, anxiety stress, and periodontal disease (11, 12). Furthermore, mental health issues have always been a significant cause of incontinence (13) and chronic illnesses also increase the prevalence of incontinence (14). Therefore, the potential relationship between MQI and UI is worth exploring in facing the enormous challenges.

The purpose of this study was to explore the relationship between MQI and UI. We hypothesized that the MQI would be negatively associated with UI in the U.S. population. By exploring this relationship, the study hopes to make a scientific contribution to UI prevention.

## 2 Methods

### 2.1 Study population information

The NHANES is a large research program evaluating the health and nutrition of the U.S. population, including both adults and children. And it has complete demographic and medical information. Secondary analysis of these data allows for the investigation of the epidemiological characteristics of diseases, which can lead to the development of appropriate health prevention policies and the strengthening of health security measures.

**Figure 1:**
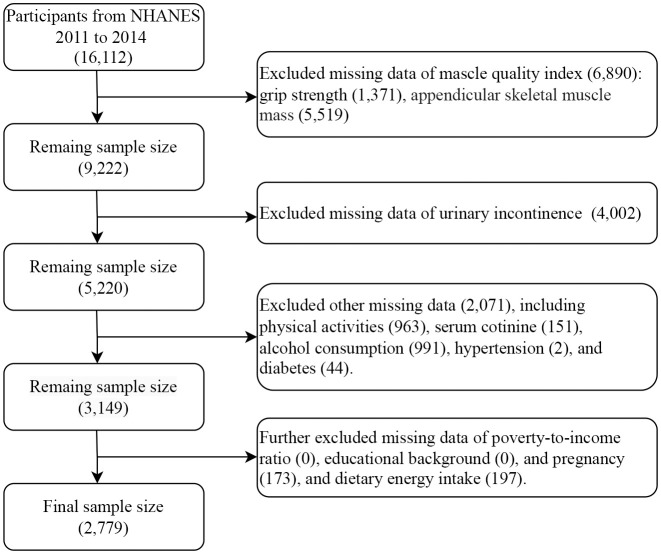
Participant screening flowchart.

This study selected the 2011-2012 and 2013-2014 survey cycles for analysis because only these two rounds contain complete data on MQI and UI. Initially, the two survey cycles included a total of 16,112 participants. First, 6,890 participants were excluded due to missing data for calculating the MQI, including grip strength (1,371) and appendicular skeletal muscle mass (5,519). Second, participants with missing data on UI (4,002) were excluded. Subsequently, participants with missing data on other variables, including physical activity (963), serum cotinine (151), alcohol consumption (991), hypertension (2), and diabetes (44) were also excluded. Finally, participants with missing data on poverty-to-income ratio (PIR) (0), educational background (0), and pregnancy (173) or dietary energy intake data (197) were excluded from the study. Finally, the sample size analyzed by the study was 2,779 (**Figure 1**).

### 2.2 Exposure definition

MQI (Kg/Kg) was defined as an exposure factor in this study. The MQI was obtained by dividing the hand-grip strength (HGS) by appendicular skeletal muscle mass (ASM). HGS was measured by a grip strength test using a handgrip dynamometer. Participants were asked to go through the test three times with each hand. HGS was the sum of the largest reading from each hand. Muscle mass is obtained by dual-energy X-ray absorptiometry. ASM was the sum of the muscle mass of the four limbs (excluding bone mineral content).

### 2.3 Outcome definition

The outcome of this study was UI. According to a previous study, urinary incontinence was defined as any one of an affirmative answer to the following three questions. 1) “During the past 12 months, have you leaked or lost control of even a small amount of urine with an activity like coughing, lifting or exercise?”. 2) “During the past 12 months, have you leaked or lost control of even a small amount of urine with an urge or pressure to urinate and you couldn’t get to the toilet fast enough?”. 3) “During the past 12 months, have you leaked or lost control of even a small amount of urine without an activity like coughing, lifting, or exercise, or an urge to urinate?”.

### 2.4 Variable selections

This study included as many covariates as possible in interviews and physical examinations of the population that could influence the MQI and UI. First, demographic factors included gender, age, race/ethnicity, educational background, and PIR. In addition, several lifestyle factors were also included as covariates in the analysis, including smoking status (serum cotinine), alcohol consumption, and physical activity. These lifestyle factors have been demonstrated to be closely associated with urinary symptoms (15–17). Physical activity was measured as metabolic equivalent score * frequency of each body activity per week * duration of each body activity (18). Two chronic diseases (hypertension, diabetes), which were associated with UI, were also included as covariates (19, 20). Finally, given the significant role of diet in the study, total dietary energy intake was included as a covariate to account for its potential influence (21).

### 2.5 Statistical analysis

Categorical variables in this study are expressed as weighted percentages. Continuous variables were tested for normal distribution and all continuous variables were non-normally distributed, the results are expressed as median (IQR). We categorized the MQI into quartiles, the lowest MQI quartiles, the lower MQI quartiles, the higher MQI quartiles, and the highest MQI quartiles. The Kruskal-Wallis test was used for non-normally continuous variables while the Rao-Scott test was used for categorical variables to compare the MQI quartile. Additionally, the corresponding effect sizes were calculated. For continuous variables, Cohen’s d was calculated, and for categorical variables, Cramer’s V was computed. Weighted univariate and multivariate logistic regression models were used to investigate the potential relationship between MQI and UI. Odds ratio (OR) and 95% confidence intervals (CI) were obtained. The model 1 was a crude model with any covariates, the model 2 was adjusted for gender and age, and the model 3 was adjusted for all covariates above. Furthermore, *P* for rends was also estimated. In addition, subgroup analyses were also performed as well as the calculation of *P* for interaction. All analyses were performed in R (4.2.2) and a *P* value less than 0.05 was deemed statistically significant.

## 3 Results

### 3.1 Characteristics of the participants

A total of 2,779 participants consisting of 1,538 males and 1,241 females were included in the final analyses (**Table 1**). The majority of the race/ethnicity was Non-Hispanic White and owned the educational level above high school. The prevalence of UI in the population was 25.45% and had a decreased trend from Q1 to Q4, as well as the prevalence of hypertension and diabetes (*P* value was less than 0.05). In addition, compared to the Q1 group, the Q4 group exhibited higher dietary energy intake, greater alcohol consumption, higher cotinine levels, and more physical activity.

**Table 1:** Characteristics of the participants in muscle quality index’ quartile.

		Q1 (<2.46)	Q2 (2.46-2.79)	Q3 (2.79-3.12)	Q4 (>=3.12)	*P* value	Effect size
	N=2,779	N=743	N=694	N=627	N=715		
Age (year)	36 [27; 47]	40 [29; 50]	39 [28; 50]	38 [28; 47]	36 [27; 46]	0.001	0.311
Gender						<0.001	0.121
Male	1,538 (54.33%)	308 (40.03%)	368 (52.01%)	362 (56.24%)	500 (69.01%)		
Female	1,241 (45.67%)	435 (59.97%)	326 (47.99%)	265 (43.76%)	215 (30.99%)		
Race/ethnicity						<0.001	0.307
Mexican American	313 (8.28%)	87 (8.78%)	84 (8.52%)	56 (6.13%)	86 (9.62%)		
Other Hispanic	224 (5.62%)	50 (5.26%)	68 (6.61%)	50 (4.88%)	56 (5.70%)		
Non-Hispanic White	1,240 (69.21%)	287 (64.22%)	310 (69.25%)	309 (74.45%)	334 (69.02%)		
Non-Hispanic Black	569 (9.83%)	244 (16.87%)	131 (8.91%)	101 (7.16%)	93 (6.36%)		
Other race	433 (7.06%)	75 (4.86%)	101 (6.70%)	111 (7.38%)	146 (9.30%)		
Educational background						<0.001	0.158
<High school	360 (9.94%)	90 (9.46%)	75 (7.91%)	64 (7.66%)	131 (14.65%)		
>High school	1,857 (70.93%)	488 (70.52%)	482 (73.43%)	452 (76.01%)	435 (63.91%)		
High school/general educational development	562 (19.13%)	165 (20.03%)	137 (18,66%)	111 (16.33%)	149 (21.44%)		
Poverty-to-income ratio						0.295	0.13
<1	571 (14.01%)	183 (17.02%)	136 (14.04%)	110 (11.80%)	142 (13.14%)		
1-3	986 (31.55%)	265 (31.66%)	249 (30.65%)	203 (29.72%)	269 (34.14%)		
>3	1,222 (54.44%)	295 (51.32%)	309 (55.30%)	314 (58.48%)	304 (52.72%)		
Hypertension						0.003	0.186
Yes	607 (21.84%)	225 (28.99%)	156 (21.87%)	107 (17.49%)	119 (18.94%)		
No	2,172 (78.16%)	518 (71.01%)	538 (78.13%)	520 (82.51%)	596 (81.06%)		
Diabetes						<0.001	0.224
Yes	164 (4.69%)	84 (8.48%)	44 (5.48%)	24 (3.17%)	12 (1.60%)		
No	2615 (95.31%)	659 (91.52%)	650 (94.52%)	603 (96.83%)	703 (98.40%)		
Urinary incontinence						<0.001	0.248
Yes	661 (25.45%)	255 (34.94%)	175 (28.26%)	121 (21.62%)	110 (16.92%)		
No	2118 (74.55%)	488 (65.06%)	519 (71.74%)	506 (78.38%)	605 (83.08%)		
Alcohol consumption	2.00 [1.00; 4.00]	2.00 [1.00; 3.00]	2.00 [1.00; 3.00]	2.00 [2.00; 4.00]	2.00 [2.00; 4.00]	<0.001	0.097
Serum cotinine	0.04 [0.01; 39.20]	0.04 [0.01; 17.60]	0.03[0.01;3.79]	0.04[0.01;28.70]	0.08 [0.02; 164]	<0.001	0.141
Dietary energy intake	1,983 [1,528; 2,566]	1,932 [1,474; 2,496]	1,981[1,575; 2,569]	1,961 [1,484; 2,642]	2,070 [1,581; 2,603]	0.033	0.089
Physical activity	2,340 [960; 5,760]	1,800 [720; 5,040]	2,400 [1,200; 5,040]	2,400 [960; 5,760]	2,640 [900; 7,520]	0.042	0.053

### 3.2 Baseline characteristics of urinary incontinence

Compared to no urinary incontinence participants, the urinary incontinence population was older, had a large proportion of Non-Hispanic White females, and was more likely to have hypertension and diabetes, less physical activity and MQI levels, and low dietary energy intake (*P* < 0.05). There were also differences in serum cotinine level and alcohol consumption (**Table 2**).

**Table 2:** Baseline characteristics of urinary incontinence/no urinary incontinence participants.

	ALL	No Urinary incontinence	Urinary incontinence	*P* value	Effect size
	N=2,779	N=2,118	N=661		
Age (year)	36 [27; 47]	35 [27; 46]	45 [35; 52]	<0.001	0.515
Gender				<0.001	1.128
Male	1,538 (54.33%)	1,417 (67.06%)	121 (17.06%)		
Female	1,241 (45.67%)	701 (32.94%)	540 (82.94%)		
Race/ethnicity				0.011	0.198
Mexican American	313 (8.28%)	241 (8.82%)	72 (6.68%)		
Other Hispanic	224 (5.62%)	169 (5.99%)	55 (4.55%)		
Non-Hispanic White	1,240 (69.21%)	927 (67.87%)	313 (73.11%)		
Non-Hispanic Black	569 (9.83%)	418 (9.63%)	151 (10.43%)		
Other race	433 (7.06%)	363 (7.69%)	70 (5.22%)		
Educational background				0.635	0.042
<High school	360 (9.94%)	280 (10.10%)	80 (9.45%)		
>High school	1,857 (70.93%)	1,416 (70.35%)	441 (72.64%)		
High school/general educational development	562 (19.13%)	422 (19.55%)	140 (17.91%)		
Poverty-to-income ratio				0.626	0.020
<1	571 (14.01%)	435 (14.45%)	136 (12.72%)		
1-3	986 (31.55%)	756 (31.47%)	230 (31.79%)		
>3	1,222 (54.44%)	927 (54.08%)	295 (55.49%)		
Hypertension				0.070	0.171
Yes	607 (21.84%)	426 (20.87%)	181 (24.68%)		
No	2,172 (78.16%)	1,692 (79.13%)	480 (75.32%)		
Diabetes				0.020	0.171
Yes	164 (4.69%)	103 (3.92%)	61 (6.92%)		
No	2,615 (95.31%)	2,015 (96.08%)	600 (93.08%)		
MQI	2.77 [2.43; 3.13]	2.84 [2.51; 3.17]	2.6 [2.30; 2.98]	<0.001	0.434
Alcohol consumption	2.00 [1.00; 4.00]	2.00 [2.00; 4.00]	2.00 [1.00; 3.00]	<0.001	0.296
Serum cotinine	0.06 [0.01; 53.9]	0.05 [0.01; 37.30]	0.03 [0.01; 39.40]	0.006	0.004
Dietary energy intake	1,970 [1,521; 2,568]	2,022 [1,542; 2,652]	1,827 [1,460; 2,318]	<0.001	0.297
Physical activity	2,400 [960; 6,000]	2,640 [1,080; 6,240]	1,680 [720; 3,840]	<0.001	0.119

### 3.3 Relationship between muscle quality index and urinary incontinence

**Table 3** demonstrated that MQI was significantly negatively correlated with UI. The OR (95%CI) in model 1 was 0.43 (0.34 to 0.55), in model 2 it was 0.65 (0.49 to 0.85), and in adjusted model 3 it was 0.67 (0.51 to 0.87). Furthermore, the OR (95%CI) were 0.94 (0.68 to 1.31), 0.71 (0.45 to 1.12), and 0.66 (0.46 to 0.94) from Q1 to Q4 in model 3, which revealed that the highest MQI group had a 34% reduction in UI compared to the lowest MQI group.

**Table 3:** Relationship between muscle quality index and urinary incontinence.

Exposure	Urinary incontinence, OR (95% CI)					
	Model 1	*P* value	Model 2	*P* value	Model 3	*P* value
MQI (Continue)	0.43 (0.34 to 0.55)	<0.001	0.65 (0.49 to 0.85)	0.002	0.66 (0.50 to 0.87)	0.006
MQI quartiles						
Q1 (<=2.46)	Reference		Reference		Reference	
Q2 (2.46-2.79)	0.73 (0.54 to 1.00)	0.053	0.92 (0.67 to 1.27)	0.605	0.95 (0.68 to 1.33)	0.733
Q3 (2.79-3.12)	0.51 (0.35 to 0.75)	0.001	0.66 (0.43 to 1.03)	0.064	0.70 (0.44 to 1.12)	0.128
Q4 (>=3.12)	0.38 (0.27 to 0.52)	<0.001	0.65 (0.45 to 0.94)	0.023	0.67 (0.46 to 0.97)	0.037
*P* for trend		<0.001		0.012		0.023

OR, odds ratio; CI, confidence interval; MQI, muscle quality index; Q1, the lowest MQI group; Q2, the lower MQI group; Q3, the higher MQI group. Q4, the highest MQI group. The model 1 was a crude model. The model 2 was adjusted for gender and age. The model 3 was adjusted for gender, age, race/ethnicity, educational level, poverty-to-income ratio, hypertension, diabetes, serum cotinine, alcohol consumption, physical activity, and dietary energy intake.

### 3.4 Subgroup analysis

**Figure 2:**
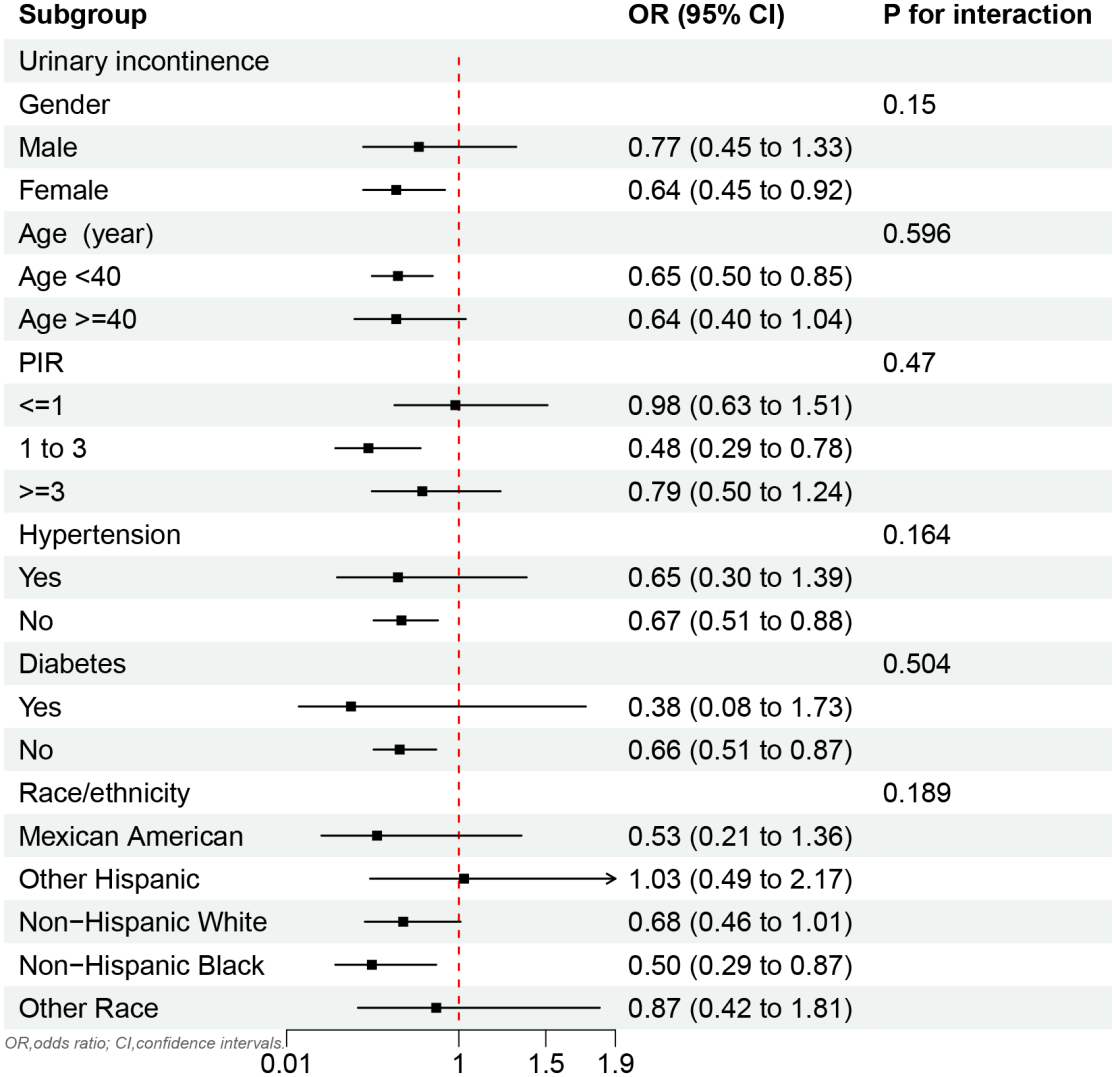
Subgroup analysis of the relationship between MQI and UI.

In subgroup analysis, the MQI was negatively associated with UI in females (OR, 0.64; 95%CI, 0.45 to 0.92), those under 40 years old (OR, 0.65; 95%CI, 0.50 to 0.85), those with PIR of 1 to 3 (OR, 0.48; 95%CI, 0.29 to 0.78), and Non-Hispanic Black (OR, 0.50; 95%CI, 0.29 to 0.87). In addition, a negative correlation was also found in some populations without chronic diseases (hypertension and diabetes) (**Figure 2**).

## 4 Discussion

### 4.1 Main results

The results found that MQI showed a significant negative relationship with UI. Not only in the crude Model 1 and Model 2, but also in the fully adjusted Model 3, the negative association between MQI and UI remained consistent and robust. The UI might be decreased by 34% with each unit increased in MQI. Moreover, this negative association persisted in females, PIR (1 to 3), those aged under 40 years, those without diabetes or hypertension, and Non-Hispanic Black. Low levels of MQI might function as a risk indicator for the UI population.

### 4.2 Comparison and interpretations

In recent years, there has been an increased focus on the impact of muscle mass on urinary tract symptoms as well as overall health. Urethral function declines by approximately 15% per 10 years. The effects of aging on the function of the urethra are obvious (22). Most importantly, aging is accompanied by loss of muscle mass and function (23). There are differences in skeletal muscle aging between females and males, with age having a more profound effect on skeletal muscle in women (24). The UI was more common in females. The impact of changes in muscle characteristics on the body has been demonstrated in certain contexts. In older men, thigh muscle strength and urinary symptoms were negatively correlated (25). Neuroelectric stimulation affecting muscle size and strength was shown to reverse the onset of hospitalized disability (26). In the future, modulation of limb muscle size and strength to regulate MQI level appears to be a viable approach to preventing UI.

The findings also suggested that MQI shows a negative correlation with UI in non-diabetic and hypertensive patients. Diabetes is associated with accelerated skeletal muscle damage (27). An overactive bladder accompanied by sympathetic excitation is evident in hypertension patients (28). Few studies have investigated the role of MQI in diabetic and hypertension patients. People with diabetes and hypertensive tend to be concerned about other, more common complications. This would be a new direction for future research. However, muscle abnormalities may have been manifested in pre-diabetes (29). Interestingly, the relationship between MQI and UI exhibits gender differences, with this association being particularly significant in females. Anatomical and hormonal differences, as well as the impact of childbirth and menopause on pelvic floor function, contribute to the increased risk of UI in females. For example, differences in sex hormones between men and women may also contribute to this phenomenon. Low estrogen levels in females have been associated with an increased risk of UI (30). Furthermore, low testosterone levels in females are also considered a risk factor for UI (31). Additionally, postmenopausal females are more susceptible to developing UI (32). Lower-income individuals often face barriers to accessing healthcare, including limited availability of preventive services and treatments for UI. Consistent with our findings, in contexts of relatively lower economic status, food insecurity is associated with a higher prevalence of sarcopenia, which in turn increases the risk of UI (33). Furthermore, higher levels of chronic stress and occupational hazards may exacerbate the relationship between UI and MQI. Among non-Hispanic Black individuals, the proportion of shift work was higher. Shift work was positively associated with urinary incontinence in women but showed no significant association in males (34). Females under financial stress reported twice as many lower urinary tract symptoms (including UI) as women without financial stress (35). Notably, participants were more likely to discuss incontinence when severe incontinence was present. Female with diabetes, the largest group of incontinence sufferers, were also reluctant to discuss diabetes. People with diabetes tended to be concerned about other, more common complications. Those with lower household incomes were less likely to discuss incontinence with the interviewer (36). However, UI appears to be prevalent among low-income older adults in other countries (37). Therefore, although we have not reached valid conclusions in the low- and high-income groups. However, attention to them cannot be reduced.

### 4.3 Strengths and limitations

First, the population included in this study was a weighted, nationally representative population. Second, the sample size of this study was large enough that the results were meaningful in the population. In addition, this study included covariates that could influence exposure factors and outcomes as much as possible, making the results more reliable.

However, the limitations were also evident. First, the outcomes were based on participants’ subjective responses, which could lead to misinformation. Second, the most significant drawback was that this study was a cross-sectional study, which did not allow for the determination of causality, but only correlation analysis. While the study provided valuable insights into MQI and UI, it was cross-sectional design limits the ability to infer causality. Longitudinal studies were necessary to determine the temporal relationship between UI and MQI. Furthermore, randomized controlled trials were essential to evaluate the effectiveness of specific interventions targeting MQI. This would help infer causality between UI and MQI and provide a robust foundation for clinical decision-making. Finally, multicenter studies involving diverse populations could enhance the generalizability and stability of the findings.

It remains unknown whether systemic muscle-strengthening training can reverse existing UI, as muscle loss and UI may interact due to various factors, including aging, gender differences, and chronic diseases. Clinically, targeted interventions to prevent muscle loss may help reduce the prevalence of UI, particularly in high-risk populations. However, it is important to note that once UI and significant muscle loss have occurred, some interventions may be insufficient to alleviate UI symptoms. Additionally, the impact of early muscle-strengthening interventions on UI prevention is an essential area of investigation.

## 5 Conclusion

In conclusion, the negative correlation between MQI and UI in this study suggests that UI seems to be prevented by changing the level of MQI. In daily life, people at risk of UI should be more muscular strength exercise thus reducing the incidence of UI. However, more prospective studies are necessary. It also provides a new perspective for the prevention of UI worldwide. However, more effective indicators related to UI should be continuously explored. By addressing these questions, future research can provide clearer guidance on the role of muscle strengthening in both the prevention and management of UI.

## Data Availability

The original contributions presented in the study are included in the article/supplementary material. Further inquiries can be directed to the corresponding authors.
